# The Fairy Tales of Science

**Published:** 1890-08-23

**Authors:** 


### The Hospital

A Weekly Journal of

Science, Medicine, Nursing, and Philanthropy.

*For Hospitals, Asylums, Medical Practitioners, Students, Nurses, Families, Parishes, Congregations, and the Charitable Public.*

Vol. VIII.—No. 204.] [August 23, 1890.

### THE FAIRY TALES OF SCIENCE.

Man has always been wonderful and fascinating to man. In every fairy story, in all history, in philosophy, in moral discourse, and in the novel and the play, man has still been the point of illumination and the active centre of interest and attention. Every fact and circumstance in the world of life gains in significance as it approaches nearer to ourselves, and loses its importance for us in proportion as it becomes more remote. Nearly a hundred years ago Rösel von Rosenhof discovered and described an animal which he then called an *Amœba.* Amœba means "change"; and von Rosenhof called his newly-discovered animal an Amœba because it never seemed to be able to maintain the same shape for more than a few minutes at a time. At first when the observer caught sight of it its shape might be almost spherical; but whilst he was still looking the creature might assume the form of an irregular lozenge; or, again, it might approximate itself to an egg or a kidney potato, or suddenly thrust out what appeared like a head; or instead of a head it might develop a leg something like that of a dining table, or two legs, or three, or half a dozen. But when the small animal came to be carefully examined it was found that it had not really a head at all, or any legs, or heart, or liver, or brain, or eyes, or nose, or ears, or even a mouth to eat with. It could eat, however, all the same, and apparently with relish. If any of its favourite articles of diet floated near it or touched it the Amœba would act exactly as if it had been a thick blanket floating in the air, into which somebody had thrown a cricket ball. A ball so thrown would in all probability be instantly enclosed in the folds of the blanket without possibility of further movement, and the blanket would fall to the ground with the ball inside it. In some such way, when a favourite article of diet touches any part whatsoever of the surface of an Amœba the whole animal changes itself into enclosing folds and keeps the food securely within them until every particle of nutriment is extracted from it. The Amœba, in short, may be regarded as consisting entirely of a universal, all-devouring mouth.

What has all this to do with man? For a long time it was not known that the Amœba had any connection with man at all beyond being an object of profound wonder as the lowest discoverable form of animal life. But more than a hundred years after Rosenhof's discovery, Beale, Max Schultze, and others, found out that the white cells of the blood of living men and women were, in almost all respects, so similar to the Amœba as to be practically identical with it. They have the same histological structure, if that may be called structure, which is practically homogeneous; the same power of changing their shapes, of eating, multiplying, and working out their destiny, as the Amœba that floats free in fresh waters or burrows in the mire of damp ground.

It was not a particularly nice idea, at least tothe peculiar intellect of the middle of the present century, that such low organisms should make our hearts and their tributary blood vessels their homes; and so far as the public is concerned, the Amœbaup to the present time has been severely let alone. Physiologists have made the best of him by pointing out that he is simply an embryo on his way to becoming a red blood corpuscle. The red blood, whose redness is due solely to its corpuscles, has been an object of interest to the world ever since Cain slew his brother Abel. Without it the maiden would be deprived of all her charming blushes, and the sanguinary novelist would be compelled to devise other methods of tragedy than that of piercing hearts with daggers, and leaving victims "weltering in their gore."

But everything comes to him who knows how to wait, and the human Amœba will probably soon receive the full reward of his extraordinary merits. Sir Joseph Lister has taken him in hand in this country, and Metchnikoff on the Continent. According to those two eminent and illustrious persons the human Amœba, or the white blood corpuscle, is in reality the brave and watchful guardian of the body. During the last few years doctors have been plunged into a state of scientific terror by their own discoveries; and the lay public has not known what to do or even to think, in the presence of the hordes of disease microbes which scientists have held up to view. Happily for us we now find that nature, which has permitted bacteria to live, has created enemies to keep them in check. It is beautiful if true; and it seems to be as true as it is beautiful. Sir Joseph Lister, at the International Medical Congress at Berlin, gave an account of the experiments of Metchnikoff, which seemed to show almost beyond a doubt that if there is anything which a human white blood corpuscle considers a *bonne bouche* it is a bacterium. When we think how long bacteria seem to have had it all their own way, so far as our knowledge of them was concerned, we cannot but feel it an immense relief to be told that the white blood corpuscles of which there are uncountable millions in the body, form a valiant garrison ready to spring upon and devour the vicious creatures as fast as they enter the human organism. Metchnikoff indeed has rechristened some of those white cells or leucocytes, and now calls them "phagocytes," that is "eating cells," instead of merely white cells. We heard long ago of the cholera bacterium, of his cousin which produces consumption, of other relations which set up scarlet fever, diphtheria, typhoid fever, and no end of diseases of the same kind, and our hair gradually rose from its posture of easy and dignified leisure, until it stood almost permanently on end. But Metchnikoff and Lister have changed all that. If a man can but retain good general health, and live in such surroundings that he shall never come in contact with really overpowering hordes of bacteria he may be quite happy. So far as the few are concerned that inhabit moderately pure air, the phagocytes, which guard our life-blood, can dispose of them with the utmost ease. It is proper to say that Koch, the bacteriologist *par excellence*, has his doubts about the "phagocyte" theory. But then Koch is not either the parent or the sponsor of it; and that is a very important fact among scientific men.

We have purposely refrained from discussing this subject in too serious or technical a way. But none the less do we feel that the active members of the medical profession, and the general public quite as much, owe a deep debt of gratitude to the laborious, conscientious, accurate, and brilliant scientists who devote their days and their great powers to researches which seem to pierce and to lay bare the profoundest mysteries of life. Only by the increase of definite knowledge is scientific progress in medicine and in human development possible. In past times our forefathers pitifully and hopelessly spent their time in beating the air when calamitous pestilences overtook them. We have knowledge, and our knowledge during the last half century has enabled us not only to rout the foe when he has appeared, but to keep him imprisoned in impassable dungeons. We shall never extirpate the last bacterium or so change the character of our blood that he will find no pabulum in it. But there can be no doubt that as science advances the forces of health will grow steadily stronger, and the deadly powers of injurious organisms will become less and less. All this we owe to the patient labours and intelligent thought of the men whom we call scientists. What reward does the great world give, of intelligent appreciation or of practical following on the lines of scientific discovery? It too often happens to men of science as it did to John the Baptist: there are voices crying in the wilderness, which men will not hear; and which, when they do hear, they do not care to understand.

